# Comparison of the Accuracy of IOL Power Calculation Formulas for Pediatric Eyes in Children of Different Ages

**DOI:** 10.1155/2020/8709375

**Published:** 2020-07-27

**Authors:** Jiaojiao Kou, Pingjun Chang, Lei Lin, Zhangliang Li, Yana Fu, Yun-e Zhao

**Affiliations:** ^1^School of Optometry and Ophthalmology, Wenzhou Medical University, Wenzhou, Zhejiang, China; ^2^Key Laboratory of Vision Science, Ministry of Health China, Wenzhou, Zhejiang, China; ^3^Department of Ophthalmology, AVIC 363 Hospital of Chengdu, Chengdu, Sichuan 610041, China

## Abstract

**Purpose:**

This study aims to compare the accuracy of five intraocular lens (IOL) power calculation formulas (SRK/T, Hoffer Q, Holladay 1, Haigis, and Holladay 2) for pediatric eyes in children of different ages.

**Methods:**

In this prospective study, patients who received cataract surgery and IOL implantation in the capsular bag were enrolled. We compared the calculation accuracy of 5 formulas at 1 month postoperatively and performed subgroup analysis with the patients divided into three groups according to their ages at the time of surgery as follows: group 1 (age ≤ 2 years, 35 eyes), group 2 (2 years < age < 5 years, 38 eyes), and group 3 (age > 5 years, 29 eyes).

**Results:**

75 patients (102 eyes) were enrolled in this study. The Haigis formula got the smallest PE among all formulas in all three groups. With regard to APE, there were no statistical differences among the formulas except group 2, with the SRK/T formula a little smaller, the Holladay 2 formula a little larger in group 1, and the Haigis formula a little smaller in group 3. In group 2, the Haigis formula had the lowest APE (0.87 ± 0.61 D), while the Holladay 2 formula had the largest (1.71 ± 1.20 D, *p* < 0.001), followed by the Holladay 1 formula (1.51 ± 1.07 D, *p*=0.002). On comparing the percentage of APE within 0.5 D and 1.0 D obtained with 5 formulas in each group, there were no statistical differences. The SRK/T formula and the Holladay 1 formula showed the highest percentage (40.00% and 60.00%) in group 1. While the Haigis formula got the highest percentage in less than 0.5 D (34.21%) and less than 1 D (60.53%) in group 2. In group 3, the Holladay 2 formula and the Haigis formula got the highest percentage less than 0.5 D (58.62%) and less than 1 D (79.31%). The multiple linear regression indicated that the age at the time of surgery was a significant factor affecting the accuracy of APE; after removing the age, AL was the only factor that affected the accuracy of APE.

**Conclusion:**

The SRK/T and the Holladay 1 formulas were relatively accurate in patients younger than 2 years old, while the Haigis formula performed better in patients older than 2.

## 1. Introduction

Primary IOL implantation has become a common and well-accepted method for optical correction in infants and young children [1]. Satisfactory refractive outcomes not only depend on successful surgical treatment but also on accurate biometrical measurement and IOL power calculation [2]. At present, all IOL power calculation formulas applied in children have been derived from studies conducted on adult eyes [3,4], children originally owning the characters of short axial length (AL), high keratometry (*K*) value, and shallow anterior chamber depth (ACD) [5], and these parameters change as children grow [6]. All these make it harder to select a proper formula to calculate IOL power [7–9]. Therefore, conducting research on different age groups was essential to compare the accuracy of formulas.

At present, most of the previous studies have a large age range and small sample size, and more importantly, very few studies have investigated specific age stages. This study aims to assess and compare the predictability of refractive outcomes using SRK/T, Hoffer Q, Holladay 1, Haigis, and Holladay 2 formulas in children of different ages who accepted cataract extraction and primary intracapsular IOL implantation.

## 2. Patients and Methods

### 2.1. Patients

All patients underwent pediatric cataract surgery with IOL implantation in the capsular bag by an experienced surgeon (Y. E. Z.) over a 5-year period (September 2013–December 2018) at the Eye Hospital of Wenzhou Medical University (Hangzhou branch), Hangzhou, China. The mean age of the patients was 41.43 months (ranging from 6 months to 84 months). This study was in accordance with the Declaration of Helsinki, and it was approved by the Office of Research Ethics, Eye Hospital of Wenzhou Medical University. Written informed consent was obtained from their parents.

### 2.2. Exclusion Criteria

Eyes with traumatic cataracts, corneal disorders such as leukoplakia, retinal diseases, IOL implantation in the ciliary sulcus or secondary implantation, severe complications, and patients who did not finish follow-up on time were excluded from the study.

### 2.3. Biometric Measurements and IOL Power Determination

For poorly cooperative patients, measurements were performed under sedation. AL and ACD were measured via an A-scan (Axis nano, Quantel Medical, France). *K* value was obtained with a handheld keratometer (Nidek Inc. Japan), with an average of at least two readings that varied <1 diopter (*D*). Horizontal corneal diameter (white to white, WTW) was measured using calipers under the surgical microscope. For cooperative children, AL, ACD, and *K* value were obtained via IOL MASTER 500 (Zeiss, Germany). For all children, lens thickness (LT) was measured by an A-scan. And we input the parameters into IOL MASTER 500 and used the built-in IOL formulas to obtain the corresponding results.

The IOL power was calculated by different formulas (with reference to http://ocusoft.de/ulib/ for constants of each formula) and was selected mainly by referring to the SRK/T, Haigis, and Holladay 2 formulas. The target diopter reference law (Table 1) was calculated according to previous studies [10,11].

### 2.4. Surgical Technique

All patients underwent surgery under general anesthesia. A 2.2 mm superior scleral tunnel incision was created followed by two 1.0 mm side incisions. Lensectomy and limited anterior vitrectomy were applied for all children; the diameter of anterior capsule was 4.5–5.0 mm, and an IOL (SA60AT/ZCB00) was implanted into the capsular bag. Finally, the main incision was closed with a 10-0 nylon suture for older children with side port incisions sealed by stromal hydration, while for younger (younger than 4-5 years old) children, all the incisions were sutured, and the side port sutures were removed 2–4 weeks after operation according to the corneal healing and the tightness of the suture.

### 2.5. Data Collection

Patients were required to attend regular clinical follow-ups, and the refraction was examined at 1 month after surgery by retinoscopy. Spherical equivalent (spherical equivalent = spherical power + cylinder power/2) was converted to calculate the prediction error (PE) as follows: PE = predicted refraction–actual refraction. And absolute PE (APE) was the absolute value of the PE. All the patients' age, sex, horizontal corneal diameter, AL, K, lens thickness (LT), IOL type and power, ACD, PE, and APE were included for accuracy analysis. The APE percentages of patients within 0.5 diopter (D), 1.0 D, and 2.0 D were calculated to determine which formula could generate the smallest error.

### 2.6. Statistical Analysis

75 patients were divided into three groups according to their age at the time of surgery: group 1 (age: ≤2 years), group 2 (>2 years and ＜5 years), and group 3 (>5 years). Data were analyzed with the statistical software SPSS 21. The Kolmogorov–Smirnov test was used to evaluate normality. The ANOVA test was used to assess intragroup difference about PE and APE results among five formulas. The chi-square test was used to analysis the different percentage of APE within 0.5 D and 1 D among five formulas. Multiple linear regression was used to evaluate the impact caused by the factors that were analyzed. *p* values lower than 0.05 were considered statistically significant.

## 3. Results

A total of 102 unilateral and bilateral eyes (75 patients; 50 males and 25 females) were enrolled in this study. The characteristics of the patients are shown in Table 2.

**Figure 1:**
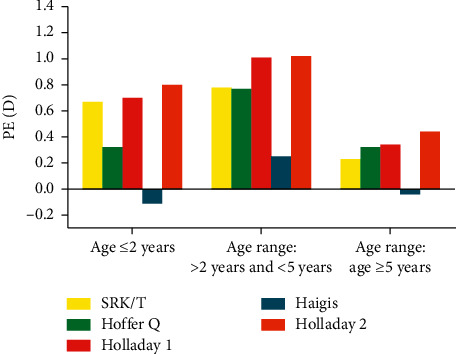
PE results of five formulas in the three study groups.

In all three groups, the Haigis formula got the smallest PE among all formulas, although there was no difference overall in the ANOVA test (Figure 1, Table 3). According to further analysis of multiple comparisons, in group 1, there was a significant difference between the Haigis (−0.11 ± 1.95 D) and Holladay 2 (0.80 ± 2.07 D) formulas (*p*=0.035, Figure 1, Table 3); in group 2, the significant difference existed between the Haigis (0.25 ± 1.04 D) and Holladay 1 (1.01 ± 1.56 D, *p*=0.014) and Holladay 2 formulas (1.02 ± 1.84 D, *p*=0.012); while in group 3, the Haigis formula got a slightly smaller PE without a significant difference.

**Figure 2:**
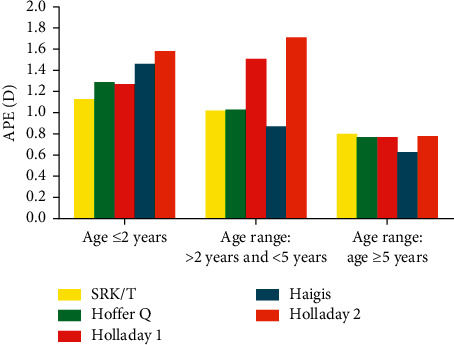
APE results of five formulas in the three study groups.

With regard to APE, there were no statistical differences overall among the formulas in the ANOVA test except group 2. According to further analysis of multiple comparisons, in group 1, the SRK/T formula got a little smaller APE and the Holladay 2 formula a little larger, while in group 3, the Haigis formula a little smaller and the SRK/T formula a little larger, without significant difference. In group 2, the Haigis formula had the lowest APE (0.87 ± 0.61 D), while the Holladay 2 formula had the largest (1.71 ± 1.20 D, *p* < 0.001), followed by the Holladay 1 formula (1.51 ± 1.07 D, *p*=0.002) (Figure 2, Table 3).

**Figure 3:**
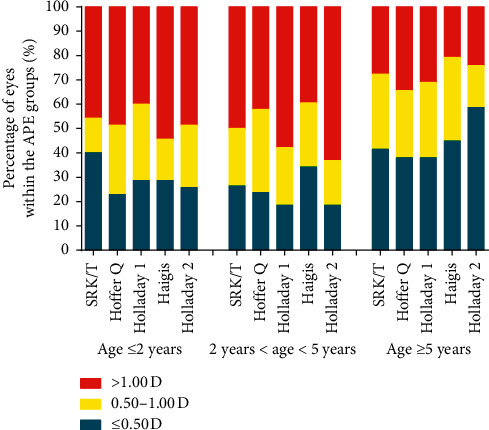
Percentage of APE of five formulas in the three study groups.

The percentage of eyes achieved the targeted absolute errors by the five formulas in the three groups is shown in Figure 3 and Table 3. In general, there was no difference among five formulas with the percentage of APE less than 0.5 D and 1 D in the three groups. In group 1, the SRK/T formula got the highest percentage (40.00%) of eyes with APE less than 0.5 D and the Holladay 1 formula got the highest percentage (60.00%) less than 1 D. In group 2, the Haigis formula got the highest percentage of APE in less than 0.5 D (34.21%) and 1 D (60.53%). In group 3, the Holladay 2 formula got the highest percentage (58.62%) of APE less than 0.5 D followed by the Haigis formula (44.83%), and the Haigis formula got the highest percentage (79.31%) less than 1 D.

Therefore, the SRK/T and the Holladay 1 formulas were relatively accurate in patients younger than 2 years old, while the Haigis formula was better in patients older than 2.

Multiple linear regression was used to evaluate the impact caused by the factors (AL, *K*, ACD, and age) that were analyzed. In cases of bilateral cataract eyes, we only analyzed the right eye. The result indicated that age was the only significant factor affecting APE (*p*=0.023). After removing the factor of age, AL, *K*, and ACD were analyzed; only AL was found to have an influence on APE (*p*=0.034).

## 4. Discussion

The eyes of younger patients have a shorter AL, a steeper cornea with a higher *K* value, and a shallower ACD [12–14]. In addition, measurements are often done imprecisely under anesthesia due to the limits of equipment, which can result in additional errors in IOL power calculation [9,15]. More important, the eye structure undergoes changes rapidly as children grow [6], the eye axial length grows rapidly and account for 90% within 2 years old, until the age of 5 years, it closes to the adult level [16]. Although the law of change is difficult to predict, we still strive to make it more precise in terms of achieving target refraction.

In the present study, in group 1, the mean AL was 20.62 mm, and the Haigis formula had the smallest PE, while the SRK/T formula got the best accuracy within 0.5 D in 40.00% and the Holladay 1 formula within 1 D in 60.00%. Kekunnaya et al. [6] conducted a study about SRK II, SRK/T, Holladay, and Hoffer Q formulas on 128 eyes of 84 children younger than 2 years (4–98 weeks); the APE of the SRK II formula (2.27 ± 1.69 D) had the smallest error, but their study had differences in the time of postoperative follow-ups. Vanderveen et al. [17] included 43 eyes with an average age of 2.5 ± 1.5 months at surgery, the mean AL was 18.1 ± 1.1 mm, and found that the Holladay 1 and the SRK/T formulas gave equally good results and had the best predictive value for infant eyes. The predicted refractions within 1.0 D by using the Holladay 1 and the SRK/T formulas were 44% and 46%, respectively. This study had similar results with ours. Neely et al. [18] studied 101 consecutive cases and reported that the SRK II, SRK/T, and Holladay 1 formulas had no significant difference in IOL power predictability in pediatric patients, while they did not further specify the number of children younger than 2 years. Most of these documents did not include the Haigis formula.

In group 2, the Haigis formula got the smallest PE and APE and achieved the best accuracy within 0.5 D in 34.21% and 1.0 D in 60.53%. Vasavada et al. [5] evaluated the Holladay 2, Holladay 1, Hoffer Q, and SRK/T formulas in 117 eyes (117 patients) with a mean age of 2.97 years and suggesting that either the SRK/T or the Holladay 2 formula could be applied for children. However, their study did not divide patients into groups based on their ages, and the age distributions in each group vary widely. Mezer et al. [19] compared the predictions of five formulas: SRK, SRK II, SRK/T, Holladay 1, and Hoffer Q. In their study, each formula missed the target refraction by approximately 1.1 D even 3.5 D –5.5 D in follow-ups of 2-3 months. Thus, they concluded that the prediction was unsatisfactory for all included formulas. Similarly, these studies also did not analyze the Haigis formula.

In group 3, the Haigis formula got the lowest PE and APE in average, while the Holladay 2 formula achieved the best prediction within 0.5D in 58.62% and the Haigis formula within 1.00D in 79.31%. Eibschitz et al. [20] conducted a spreadsheet program with AL (16 to 28 mm) and *K* (40 to 55 D) as input parameters to predict IOL power and reported that the Holladay 1 and Haigis formulas were highly accurate with similar performance in IOL prediction. O'Gallagher et al. [21] enrolled 46 patients (67 eyes) undergone surgery younger than eight (mean 3.8 years) and analyzed the Hoffer Q, Holladay 1, SRK II, and SRK/T formulas prediction, and their results indicated that the SRK/T formula was the most accurate formula.

For patients younger than 2, more mistakes may occur in examinations due to the poor cooperation comparing other groups. The accuracy of the Haigis formula seemed not very satisfying, and the SRK/T and Holladay I formulas got better results. While for patients older than 2, the Haigis formula performed better. The possible reason is that the Haigis formula can predict better the effective position of IOL, although it may need further study, and the a1 constant of the Haigis formula is tied to the measured ACD, which can be an issue in children of different eye dimensions [22]. The Holladay 2 formula needs ACD as well as lens thickness for calculation, while lens thickness may vary due to lens abnormality such as posterior capsular defect [23]. Therefore, it is important to realize the differences between children's eyes and adults' eyes [24].

Multiple linear regression analysis demonstrated that age was a major factor affecting APE. Li et al. [25] obtained the similar result with us. Ramesh Kekunnaya et al. [6] reported that, within this age group, age did not influence the absolute PEs for any of the formulas. We removed the age at the time of surgery and found that AL was the only factor that has an effect on APE. Vander Veen et al. [17] also reported that the AL is the only factor that affects the accuracy of the prediction results; thus, their finding was similar with ours.

The present study has some limitations. First, we checked the eye parameters and retinoscopy under sedation for poor cooperative children, and measurement errors might occur. Second, A-scan and IOL MASTER 500 were both applied to examinations, and errors may be produced by different apparatus.

Despite the limitations, the present study had some advantages in dividing the children into three different age groups and analyzing the accuracy of five formulas. In conclusion, our results show that the SRK/T and Holladay 1 formulas were relatively accurate in patients younger than 2 years old, while the Haigis formula performed better in patients older than 2.

## Figures and Tables

**Table 1:** Target diopter reference law.

Age (years)	Target diopter (D)
<1	+6.00
1.5	+4.00
2	+3.00
3	+2.50
4	+2.00
5	+1.50
6	+1.00
7	0.00

**Table 2:** Patients demographic characteristics (*n* = 102 eyes).

	Age ≤ 2 years (*n* = 35)	>2 years and ＜5 years (*n* = 38)	Age ≥ 5 years (*n* = 29)

Average age in month (range)	17.63 (6–24)	40.33 (24–54)	71.59 (60–84)
No. of eyes	35	38	29
Average AL in mm (range)	20.62 (18.14–23.81)	21.96 (19.44–25.85)	23.00 (20.62–25.63)
Average K in diopter (range)	44.55 (40–51.25)	43.96 (41.23–49.81)	44.26 (41.98–49.25)
Average ACD in mm (range)	2.97 (2.03–4.5)	3.05 (2.26–3.73)	3.42 (2.20–4.83)
Average WTW in mm (range)	10.26 (9.10–11.50)	10.85 (9.8–12.40)	11.52 (10.50–12.50)
Average IOL power in diopter (range)	23.64 (17–30)	22.87 (12–28)	20.84 (7–28)

**Table 3:** Refractive outcomes of all the pediatric patients using five intraocular lens power calculation formula.

	Age ≤ 2 years		>2 years and <5 years		Age ≥ 5 years	
	PE ± SD	APE ± SD	PE ± SD	APE ± SD	PE ± SD	APE ± SD
*Error (D)*						
SRK/T	0.67 ± 1.57	1.13 ± 1.27	0.78 ± 0.97	1.018 ± 0.70	0.23 ± 1.04	0.80 ± 0.70
Hoffer Q	0.32 ± 1.70	1.29 ± 1.14	0.77 ± 0.97	1.03 ± 0.69	0.32 ± 0.93	0.77 ± 0.60
Holladay 1	0.70 ± 1.66	1.27 ± 1.26	1.01 ± 1.56	1.51 ± 1.07	0.34 ± 0.93	0.77 ± 0.61
Haigis	−0.11 ± 1.95	1.46 ± 1.27	0.25 ± 1.04	0.87 ± 0.61	−0.04 ± 0.82	0.63 ± 0.50
Holladay 2	0.80 ± 2.07	1.58 ± 1.54	1.02 ± 1.84	1.71 ± 1.20	0.44 ± 0.92	0.78 ± 0.65
*p* value	0.196^a^	0.630^a^	0.084^a^	<0.001^a^	0.360^a^	0.854^a^

*Percentage (%)*						
	<0.5	<1	<0.5	<1	<0.5	<1
SRK/T	40	54.29	26.32	50	41.38	72.4
Hoffer Q	22.86	51.43	23.68	57.89	37.93	65.52
Holladay 1	28.57	60	18.42	42.11	37.93	68.97
Haigis	28.57	45.71	34.21	60.53	44.83	79.31
Holladay 2	25.71	51.43	18.42	36.84	58.62	75.86
*p* value	0.578^b^	0.824^b^	0.469^b^	0.185^b^	0.483^b^	0.786^b^

SD, standard deviation; PE, prediction error; and APE, absolute postoperative refraction. ^a^ANOVA test. ^b^Chi-square test.

## Data Availability

The data used to support the findings of this study are available from the corresponding author upon request.
